# Nanoplatforms for Irinotecan Delivery Based on Mesoporous Silica Modified with a Natural Polysaccharide

**DOI:** 10.3390/ma15197003

**Published:** 2022-10-09

**Authors:** Ana-Maria Brezoiu, Ana-Maria Prelipcean, Daniel Lincu, Mihaela Deaconu, Eugeniu Vasile, Rodica Tatia, Ana-Maria Seciu-Grama, Cristian Matei, Daniela Berger

**Affiliations:** 1 Faculty of Chemical Engineering and Biotechnologies, University “Politehnica” of Bucharest, 1-7 Gheorghe Polizu Street, 011061 Bucharest, Romania; 2 National Institute of R&D for Biological Sciences, 296 Splaiul Independetei, 060031 Bucharest, Romania; 3 “Ilie Murgulescu” Institute of Physical Chemistry, Romanian Academy, 202 Splaiul Independentei, 060021 Bucharest, Romania

**Keywords:** ulvan, mesoporous silica, natural polysaccharide, irinotecan, cytostatic agent, drug delivery systems

## Abstract

Natural compounds are an important source of beneficial components that could be used in cancer therapy along with well-known cytostatic agents to enhance the therapeutic effect while targeting tumoral tissues. Therefore, nanoplatforms containing mesoporous silica and a natural polysaccharide, ulvan, extracted from *Ulva Lactuca* seaweed, were developed for irinotecan. Either mesoporous silica-ulvan nanoplatforms or irinotecan-loaded materials were structurally and morphologically characterized. In vitro drug release experiments in phosphate buffer solution with a pH of 7.6 emphasized the complete recovery of irinotecan in 8 h. Slower kinetics were obtained for the nanoplatforms with a higher amount of natural polysaccharide. Ulvan extract proved to be biocompatible up to 2 mg/mL on fibroblasts L929 cell line. The irinotecan-loaded nanoplatforms exhibited better anticancer activity than that of the drug alone on human colorectal adenocarcinoma cells (HT-29), reducing their viability to 60% after 24 h. Moreover, the cell cycle analysis proved that the irinotecan loading onto developed nanoplatforms caused an increase in the cell number trapped at G0/G1 phase and influenced the development of the tumoral cells.

## 1. Introduction

An ideal drug delivery system for anticancer agents should be designed to significantly improve the current treatments that use the drug, preventing its degradation and ensuring a higher concentration in tumors. Moreover, the carrier should be designed to ensure selectivity and/or accumulation of the cytostatic agent in cancer cells and hence, to reduce its toxicity on healthy cells [1].

In the last period, research has been focused on the valorization of natural compounds that are superior to synthetic substances while achieving good biological effects. Marine polysaccharides are considered biomaterials exhibiting several important characteristics. They have zwitterionic nature due to both positively (NH_3_^+^) and negatively (COO^−^, SO_3_^−^) charged groups acting as polyelectrolytes. They contain glycosidic bonds that can break easily under hydrolase action and thus, an improved biodegradability is achieved [2,3].

Moreover, polysaccharides could be used to target cancer cells (of the liver [4], breast tissue [5], epithelial cells or macrophages). Sulfonated polysaccharides are internalized in cancer cells based on their negative charge and interact with mannose receptors that additionally recognize fucose and *N*-acetylglucosamine residues [3]. Not only polysaccharides but also cyclic oligosaccharides (cyclodextrins) are widely used for targeting tumor cells [6].

Irinotecan, a well-known cytostatic, is poorly water-soluble, especially at neutral or basic pH. Therefore, it is a challenge to develop drug delivery systems for oral administration with a high amount of the recovered drug. The literature data on irinotecan encapsulation and release show that, in most cases, the cytostatic agent is partially recovered from the carriers after a long period of time. Several studies were reported for the incorporation of irinotecan and its derivatives or metabolites (SN-38) to achieve reduced toxicity and targeted action. Iliescu et al. proposed systems for incorporating irinotecan into montmorillonite and montmorillonite-alginate composite materials from which the cytostatic agent was gradually released (7–10%(w/w)) and the composite system could be recommended for intraoperative chemotherapy of colorectal carcinomas [7]. The entrapment of irinotecan in liposomes was reported to ensure targeted delivery of an antineoplastic agent and the system was tested on mouse xenograft for the possible treatment of breast cancer [8]. Moreover, glutathione redox-activated systems were proposed to improve the cellular absorption of irinotecan [9]. Wu et al. reported the use of superparamagnetic carriers containing chitosan and Fe_3_O_4_ nanoparticles for irinotecan (15%(w/w)) that can be magnetically directed to the target site [10]. Functionalized mesoporous silica has also been reported for irinotecan encapsulation. The drug was completely released in 5–7 h in phosphate buffer solution (PBS) pH 7.4 proving an improved efficacy compared to the drug alone in tests on mouse embryonic fibroblasts [11].

Mesoporous silica, a biocompatible material [12,13], is used due to its high porosity, tailored pore size and surface properties in various drug delivery applications [14] for antifungal agents [15], antibiotics [16,17,18,19,20,21], natural compounds [22,23,24,25,26,27], cytostatic agents [16,28,29], etc., to control the release process of biologically active compounds.

It is well known that many drugs are very hydrophobic having poor water solubility, and thus, low bioavailability. Due to the nanoconfinement effect, the encapsulation of active pharmaceutical ingredients (API) in an amorphous state into the silica mesopores represents a strategy to enhance their aqueous solubility and dissolution rate in comparison with that of the crystalline API, and therefore, their bioavailability [30,31].

Well-designed drug delivery systems containing mesoporous silica with tailored surface properties can enable controlled delivery of anticancer agents targeting diseased sites, reducing side effects that occur during conventional therapies [32]. The coating of mesoporous silica with a polymer has many advantages. It can provide sensitivity to different stimuli (pH, light, temperature, glutathione, etc.) triggering the delivery of the encapsulated drug molecules into silica mesopores in the desired environment. The chemical modification of the silica surface plays a crucial role in the success of the coating procedure because the best results are achieved when electrostatic interactions between silica and polymer are established [33].

Moreover, polymer-silica hybrid aerogels with large porosity can be employed in either bone tissue regeneration or drug delivery applications. It was reported that silica aerogels dispersed in the chitosan network presented bone bioactivity and good mechanical properties. Dextran or dextran-aldehyde coated silica aerogels, whose surface was modified with (3-aminopropyl) triethoxysilane, were used as carriers for 5-flurouracil. The polymer’s role was to target the tumoral tissue and trigger the delivery of the anticancer agent [34]. The silica aerogels present larger pores and higher specific surface area and pore volume than mesoporous silica being able to accommodate higher amounts of the drug or larger molecules. However, in the case of mesoporous silica, it is possible to tailor the pore diameter during its synthesis and to achieve drug amorphization due to the nanoconfinement effect [30].

Considering that the surface modification of aminopropyl-functionalized mesoporous silica with ulvan could improve the irinotecan delivery, while targeting tumoral tissue, herein we report for the first time the use of ulvan extracted from *Ulva lactuca* green seaweed to develop mesoporous silica-ulvan nanoplatforms for irinotecan.

## 2. Materials and Methods

### 2.1. Materials

For mesoporous silica supports synthesis and drug delivery experiments, tetraethyl orthosilicate (TEOS, Fluka, Seelzer, Germany), poly(ethylene glycol)-block-poly(propylene glycol)-block-poly(ethylene glycol)—Mw = 5800 (Pluronic, P123, Aldrich, Aldrich Chemical Co Inc., Milwaukee, WI, USA), trimethyl-hexadecylammonium bromide (CTAB, Alfa Aesar, Ward Hill, MA, USA), hydrochloric acid 37% (Sigma-Aldrich Co., Merck Group, Darmstadt, Germany), 25%(w/w) ammonia aqueous solution (Scharlau, Scharlab S.L., Barcelona, Spain), aminopropyl triethoxysilane (APTES, Tokyo Chemical Industry, TCI, Tokyo Japan), N-Buthyldiethanolamine (BuDEA, Sigma), absolute ethanol (Sigma-Aldrich), toluene (Riedel de Haen, Honeywell Riedel-de Haën, Seelzer, Germany), acetone (Sigma, Merck Group, Darmstadt, Germany), Irinotecan hydrochloride trihydrate (Iri, TCI), potassium dihydrogen phosphate (KH_2_PO_4_, Merck, Merck Group, Darmstadt, Germany), sodium hydrogen phosphate (Na_2_HPO_4_, Sigma–Aldrich), sodium chloride (NaCl, Sigma-Aldrich) and potassium chloride (KCl, Sigma-Aldrich) were used as received without further purification. Ultrapure deionized water (Millipore Direct-Q3UV water systems product no. C9185, Merck Group, Darmstadt, Germany with Biopack UF cartridge) was used in all experiments.

### 2.2. Methods

#### 2.2.1. Ulvan Extract Preparation and Characterization

Ulvan was extracted from *Ulva lactuca* seaweed (Black Sea). The green algae were treated with acetone to remove chlorophyll and then with methanol (1/5 vegetal material/solvent ratio, g/mL) to remove polyphenolic compounds. After each extraction stage, the algal residue was isolated by centrifugation (6000 rpm/10 min) and dried at room temperature overnight. To obtain the ulvan extract, the algal residue was heated under reflux for 3 h and the solid was isolated through centrifugation. The supernatant was evaporated using a rotary evaporator (RE100-Pro, DLAB Scientific, Beijing, China) at 60 °C in dynamic conditions (150 rpm) until it reached a quarter of its volume. Then, the supernatant was precipitated three times in absolute ethanol to obtain the ulvan extract. The resulting solid was filtered off and then dried by lyophilization (Martin Christ Alpha 2-4 LSCbasic, Martin Christ Gefriertrocknungsanlagen GmbH, Osterode am Harz, Germany).

The acetonic and methanolic fractions were analyzed regarding their chlorophyll and total polyphenolic content by methods that we previously reported [27,35]. The ulvan extract was characterized by the total carbohydrate content determination, wide-angle X-ray diffraction (XRD, Rigaku MiniFlex II diffractometer, Rigaku Corporation, Tokyo, Japan), Fourier Transform infrared spectroscopy (KBr pellet technique, FTIR, Bruker Tensor 27, Bruker Corporation Optik GmbH, Bremen, Germany), thermo-gravimetric analysis coupled with differential thermal analysis (TG-DTA, GA/SDTA851e from Mettler Toledo, Greifensee, Switzerland), differential scanning calorimetry (DSC, Mettler Toledo DSC823e calorimeter), as well as scanning electron microscopy (SEM, Tescan Vega 3 LMH electron microscope equipped with energy dispersive X-ray detector, Brno, Czech Republic).

#### 2.2.2. Synthesis of Mesoporous Silica-Type Supports

For obtaining SBA-15 mesoporous silica, Pluronic P123 triblock copolymer as a structure-directing agent and tetraethyl orthosilicate (TEOS) as silica source were used. The details of SBA-15 silica were presented elsewhere [36,37].

MCM-41B silica was also prepared by sol-gel method using CTAB and ammonia as structure-directing agent and catalyst, respectively. To the CTAB aqueous solution in which the corresponding 25%(w/w) NH_3_ aqueous solution volume was added, a mixture of TEOS and *N*-buthyldiethnolamine (BuDEA) was poured dropwise using a molar ratio of TEOS/CTAB/BuDEA/NH_3_/H_2_O = 1.000/0.146/0.200/28.113/148.820. The reaction mixture was kept under magnetic stirring at 40 °C for 24 h. Then, the reaction mixture was hydrothermally treated at 150 °C for 24 h. The solid was filtered off, washed with warm water, and dried at room temperature. The sample was denoted MCM-41B precursor. To obtain MCM-41B material, the precursor was subjected to an extraction process in saturated NH_4_Cl solution at 60 °C, for 1 h in an ultrasonic bath (Bandelin Sonorex Digitec ultrasonic bath—Berlin, Germany) and then the solid was filtered off, washed with water, dried at room temperature, and calcined at 550 °C, 5 h.

Mesoporous silica functionalized with aminopropyl groups (samples denoted SBA-NH_2_ and MCMB-NH_2_) was obtained by the post-grafting approach using the calcined silica samples (SBA-15 or MCMB-41) and (3-aminopropyl) triethoxysilane (APTES). The post-grafting approach was selected for silica functionalization because in the case of this method the organic groups are mainly bound to the particle’s surface. The positively charged amine groups favor the retention of ulvan through electrostatic interactions. Moreover, the post-grafting approach better preserves the morphology of the silica particles, but the dispersion of the organic groups is usually less uniform than in the case of the co-condensation method [38]. The functionalization reaction was performed in toluene (molar ratio of APTES/silica/toluene = 1/5/235) for 15 h, under reflux, in inert conditions. Then, the solids were separated by centrifugation, washed with toluene, acetone, ethanol, and finally with 1M HCl aqueous solution and then dried at room temperature.

**Figure 1:**
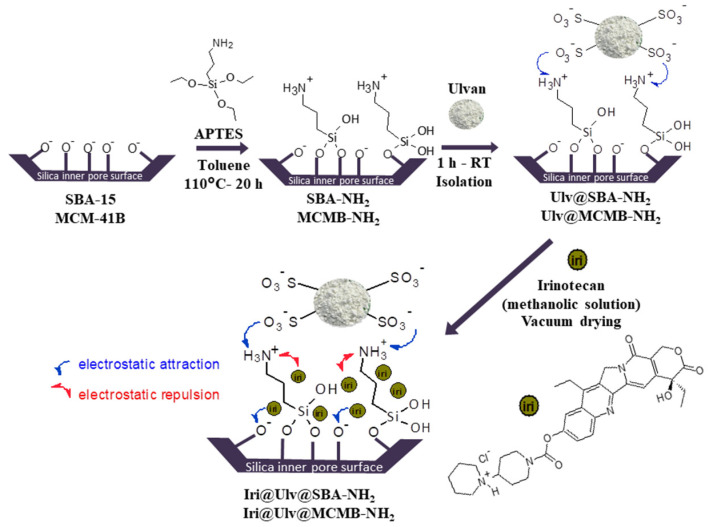
Synthesis of silica-ulvan nanoplatforms and irinotecan-loaded samples.

Mesoporous silica−ulvan nanoplatforms were obtained by putting in contact ulvan aqueous solution (3.5 mg/mL) with functionalized mesoporous silica (mass ratio of silica/ulvan = 4/1) for 1 h on an orbital shaker (Grant bio-PSU-10i, Grant Instruments, Cambridge, United Kingdom) at 150 rpm. Afterwards, the solid was isolated by filtration and dried at room temperature (Figure 1). The solids were denoted Ulv@silica-NH_2_.

#### 2.2.3. Drug-Loaded Samples Preparation

The irinotecan-loaded samples were obtained by incipient wetness impregnation method [16,39] using a methanolic solution of the drug (9 mg/mL). In brief, over 100 mg carrier (previously outgassed under vacuum for 4 h) the corresponding volume of the freshly prepared irinotecan methanolic solution was added and the resulting suspension was homogenized on an orbital shaker for 1 h (150 rpm). Then, the suspension was dried in a vacuum, in dark conditions, until complete drying was achieved. The obtained solids were denoted Iri@Ulv@silica-NH_2_.

#### 2.2.4. Irinotecan Release Experiments

The irinotecan release experiments were performed using a concentration of 0.1 mg/mL irinotecan, in simulated colon cellular medium pH 7.6 (8.01 g/L NaCl, 0.2 g/L KCl, 1.42 g/L Na_2_HPO_4_, 0.27 g/L KH_2_PO_4_) [40], under constant magnetic stirring (100 rpm). A dialysis tubing cellulose membrane bag (Sigma-Aldrich, cutoff 14,000, Merck Group, Darmstadt, Germany), containing the corresponding amount of drug-loaded sample was suspended in 1 mL PBS just before the experiment and then immersed in 90 mL PBS. At predetermined time intervals, aliquots of the release medium were withdrawn and analyzed by UV–vis spectrometry (Shimadzu UV-1800 spectrophotometer, Shimadzu Corporation, Kyoto, Japan). The drug release amount (μg Iri/mL) was calculated based on a calibration curve (y = 0.0584 ∗ x, R^2^ = 0.9993) recorded at 255 nm wavelength.

#### 2.2.5. Materials Characterization

The FTIR spectra were recorded in the range of 4000–400 cm^−1^ wavenumber (KBr pellets technique) on a Bruker Tensor 27 spectrophotometer (Bruker Corporation Optik GmbH, Bremen, Germany) to confirm the presence of the cytostatic agent. Small- and wide-angle powder X-ray diffraction analyses were performed using a Rigaku MiniFlex II diffractometer (Rigaku Corporation, Tokyo, Japan) using Cu-Kα radiation in the range of 1.2–6.0 ° (2θ) and 10–60 ° (2θ), respectively, with a scanning speed of 0.5 °C/ min with 0.01 °/step.

The content of functional groups linked on silica surface and irinotecan from the drug-loaded samples was determined using DTA-TG analysis performed in air, in the temperature range of 25–800 °C, using a Mettler Toledo GA/SDTA851e (Greifensee, Zürich, Switzerland) equipment under synthetic air flow.

Differential scanning calorimetry (DSC) analysis was performed under a N_2_ atmosphere using a Mettler Toledo DSC3 calorimeter (Greifensee, Zürich, Switzerland), at a heating rate of 5 °C/min, under 80 mL min^−1^ nitrogen flow and pierced crimped aluminum pans to evaluate the ulvan extract stability and the state in which irinotecan was present into nanoplatforms. Melting temperatures were computed as onset temperatures.

The morphology of carriers was investigated by scanning electron microscopy (SEM) on a Tescan Vega 3 LMH microscope coupled with an energy dispersive X-ray (EDX) detector and transmission electron microscopy (TEM, Hillsboro, OG, USA) TECNAI F30 G^2^ S-TWIN high-resolution transmission electron microscope equipped with a field emission electron gun and a maximum accelerating voltage of 300 kV.

For the textural parameters (specific surface area, *S*_BET_, calculated through Brunauer–Emmett–Teller method in the 0.05–0.25 relative pressure range, the total pore volume, *V*_p_, measured at 0.99 relative pressure, and the average pore diameter, *d_BJH_*, computed by Barrett–Joyner–Halenda model from the corresponding isotherm adsorption branch) of the pristine and functionalized silica samples, the nitrogen adsorption–desorption isotherms (Autosorb iQ_2_ gas sorption analyzer, Quantachrome Instruments, Boynton Beach, FL, USA) were recorded.

#### 2.2.6. Biological Evaluations of Ulvan Extract and Irinotecan-Loaded Samples

The biocompatibility evaluation of ulvan extract was performed on mouse fibroblasts from NCTC clone L929 cell line (ECACC, Sigma-Aldrich, Germany), cultured in Minimum Essential Medium (MEM) supplemented with 10%(*v*/*v*) fetal calf serum (FCS), 2 mM L-glutamine and 1%(*v*/*v*) antibiotic mixture (penicillin-streptomycin-neomycin), in a humidified atmosphere with 5% CO_2_, at 37 °C, until subconfluence. The cells morphology was evaluated by optical microscopy (Zeiss Axio Observer 20X, Jena, Germany). L929 cells were stained with Giemsa reagent (Sigma–Aldrich Co., Merck Group, Darmstadt, Germany) and treated with 2 mg/mL or 3 mg/mL polysaccharide extract and incubated for 48 h.

The antiproliferative potential of irinotecan, nanoplatforms and irinotecan-loaded nanoplatforms was assessed on human colon cancer cell line HT-29 (ATCC, manufacturer, Darmstadt, Germany) cultured in Dulbecco’s Modified Eagle Medium (DMEM, Sigma-Aldrich Co., Darmstadt, Germany) supplemented with 20%(*v*/*v*) fetal calf serum (FCS, Sigma-Aldrich Co., Merck Group, Darmstadt, Germany), 2 mM L-glutamine and 1%(*v*/*v*) antibiotic mixture (penicillin-streptomycin-neomycin), in a humidified atmosphere with 5% CO_2_, at 37 °C, until subconfluence.

Stock suspensions of tested samples (nanoplatforms and irinotecan-loaded carriers) were prepared in a culture medium at a concentration of 1 mg/mL by incubation at 37 °C for 24 h and then were filtered through 0.22 µm membrane filters (Millipore, Merck Group, Darmstadt, Germany).

Prior to evaluation, cells were seeded in 96-well microplates, at a density of 4 × 10^4^ cells/mL and allowed to adhere by incubation at 37 °C in a humidified atmosphere with 5% CO_2_, for 24 h. Then, the culture medium was replaced with a fresh medium containing different concentrations of samples ranging from 1–250 μg/mL. The plates were incubated at 37 °C under standard conditions, for 24 h and 72 h, respectively. Cells incubated in a culture medium without a sample were used as control.

The antiproliferative effect was assessed by MTT colorimetric assay that evaluates cell metabolic activity, respectively, the capacity of enzymes from viable cells to reduce the tetrazolium dye MTT, 3-(4,5-dimethylthiazol-2-yl)-2,5-diphenyltetrazolium bromide, to its insoluble formazan (purple color). Briefly, at the end of each incubation period, the culture medium was removed from each well and the cells were incubated with 50 μg/mL MTT solution, at 37 °C, for 3 h. The insoluble formazan crystals were dissolved with isopropanol and then, the absorbance was recorded at 570 nm using the microplate reader Mithras LB 940 (Berthold Technologies GmbH & Co.KG, Bad Wildbad, Germany). The amount of formazan was directly correlated to the number of metabolically active cells. The results were reported as a percentage relative to the control culture, considered 100% viable and the effective concentration (EC50) was selected as the drug concentration that decreased 50% of the cellular viability.

HT-29 cells were seeded at a cell density of 6 × 10^4^ cells/mL in 12-well culture plates and then were maintained in McCoy culture medium (supplemented with 10% FBS and 1% antibiotics) at 37 °C in a humidified atmosphere with 5% CO_2_. At 24 h after seeding, the cells were incubated in the presence of tested samples at concentrations of 100 and 250 μg/mL.

To analyze cell-cycle progression, cells were harvested after 48 h and fixed in 70% ethanol overnight at 4 °C, followed by washing with phosphate buffer solution (PBS). Then, the cells were incubated with RNase (10 μg/mL) and resuspended in propidium iodide (50 μg/mL). The cell cycle analysis was performed using a Becton Dickinson LSR II flow cytometer (BD Biosciences, Franklin Lakes, NJ, USA) and the cellular DNA content was quantified using ModFit LT 3.0 software.

## 3. Results and Discussion

### 3.1. Characterization of Ulvan, Silica-Ulvan Nanoplatforms and Irinotecan-Loaded Samples

#### 3.1.1. Characterization of Pretreatment Fractions and Ulvan

The total polyphenols content (TPC) of methanolic extract from *Ulva lactuca* seaweed was determined based on a calibration curve for gallic acid [35] and the average values ± standard deviation are listed in Table 1. The methanolic extract had a higher content of polyphenols than that reported by El-Baky et al. [41] for dichloromethane-methanol extract (21.3–34.1 mgCHt/g extract). However, the TPC value of the methanolic fraction is significantly lower than the values reported by El-Boukary et al. (54.1–112.6 mg GAE/g extract) [42]. One can observe that in methanol, the more polar solvent chlorophyll-b was better extracted than chlorophyll-a. The last was in a higher amount in acetonic fraction.

The total carbohydrate content was determined for hydrolyzed ulvan sample using a freshly prepared anthrone-sulfuric acid reagent. The ulvan extract had a high amount of carbohydrates (483 ± 47 mg glucose equivalent/g extract). This content is slightly higher than the average value reported by Kidgell et al. (37.7%(w/w)) [43].

**Figure 2:**
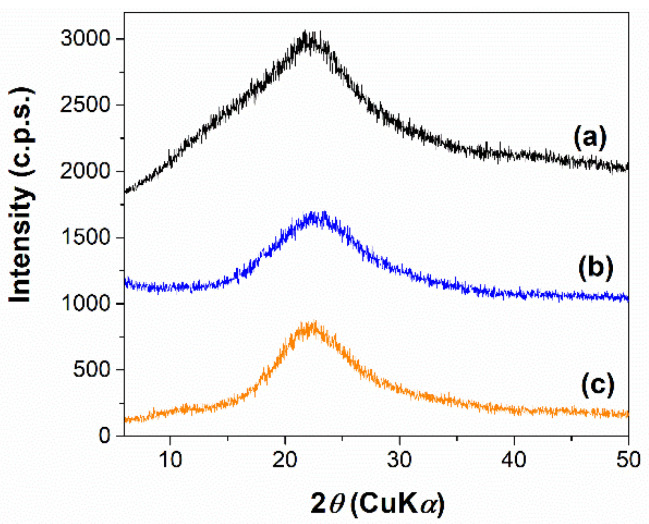
Wide-angle X-ray diffraction patterns of ulvan extract (**a**), Iri@Ulv@SBA-NH_2_ (**b**) and Iri@Ulv@MCMB-NH_2_ (**c**).

**Figure 3:**
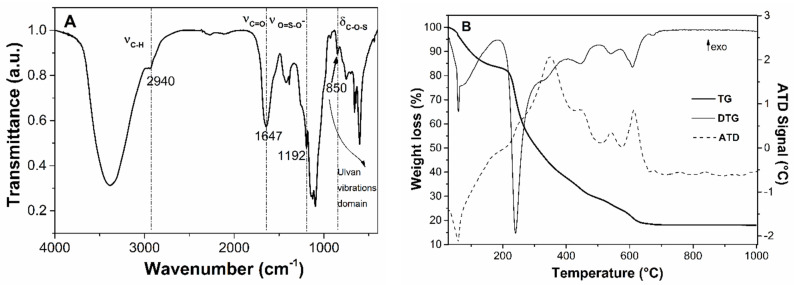
Fourier Transform Infrared spectrum (**A**) and thermogravimetric analysis (**B**) of ulvan extract.

Wide-angle XRD patterns of the ulvan extract demonstrate the obtaining of an amorphous polysaccharide extract (Figure 2—curve a). The FTIR spectrum (Figure 3A) allows the identification of several bands: stretching vibrations of hydroxyl groups in 3000–3500 cm^−1^ domain, C-H asymmetric stretching vibrations at 2940 cm^−1^ [44], carboxyl group band of uronic acids at 1647 cm^−1^, very intense asymmetric stretching vibrations of ether-glycosidic bonds at 1240 cm^−1^, and asymmetric stretching vibration of the sulphate group in the range of 1140–1050 cm^−1^. However, the most important region of the FTIR spectra is the 600–850 cm^−1^ domain, assigned to skeleton vibrations of C-O-S bonds of ulvan sulphonate groups [44].

Based on thermogravimetric (TG) analysis (Figure 3B) of the ulvan extract, a residue containing inorganic compounds (18.1%(w/w)) was obtained [45].

The differential scanning calorimetry (DSC) analysis of the ulvan extract was also performed. In the first heating cycle, an endothermic event was observed associated with humidity evaporation, which no longer occurred in the following heating cycles [45]. The absence of a strong endothermic event in the second heating cycle proves that the ulvan extract does not melt before it decomposes, or the melting point is close to the decomposition temperature (Supplementary Materials, Figure S1).

SEM investigation coupled with energy dispersive X-ray analysis (Supplementary Materials, Figure S2) highlighted the presence of sulfur and several other elements of the ulvan. S/metals (Mg, Ca, K and Na) and S/Cl molar ratio values of 0.588 and 1.739, respectively, are calculated from the EDX spectrum (Figure S2A). The SEM image with EDX elemental mapping (Supplementary Materials, Figure S2B) showed that sulfur from sulphonate groups is uniformly distributed in the ulvan extract.

#### 3.1.2. Characterization of Silica-Ulvan Nanoplatforms and Irinotecan-Loaded Samples

**Figure 4:**
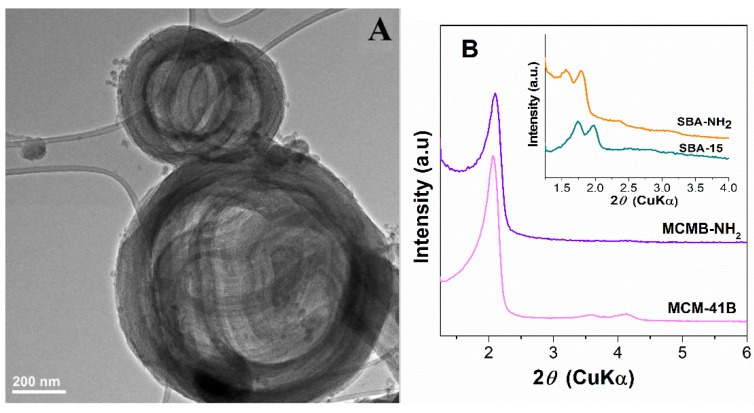
TEM image of MCM-41B sample (**A**) and small-angle XRD patterns of mesoporous SBA-15 silica and after aminopropyl groups grafting on silica, SBA-NH_2_ (**B**).

The synthesis of MCM-41B material was performed using an optimum TEOS/*N*-BuDEA molar ratio to obtain very porous ball-like structures without a loss of the pore framework ordering. Various TEOS/N-BuDEA molar ratios, 2.5/1, 5/1 and 7.5/1, were used in the synthesis of MCM 41-type silica and the morphology was evaluated by TEM microscopy. One can observe that the TEOS/N-BuDEA ratio used in the synthesis influenced the formation of ball-like structures. A low molar ratio allowed the formation of very porous ball-like structures (Supplementary Material, Figure S3A), but induced a less ordered pore array and lower pore size and specific surface area values (Supplementary Material, Figure S4, Table S1). A high molar ratio suppressed the formation of porous ball-like structures and determined a decrease in pore volume and specific surface area values (Supplementary Material, Table S1). Hence, the optimum TEOS/N-BuDEA ratio was 5/1, which was used for the synthesis of MCM-41B material. TEM investigation of the MCM-41B sample (Figure 4A) revealed highly porous ball-like structures (in the range of 400–1000 nm) consisting of long, twisted channels of mesopores and an ordered hexagonal pore array characteristic for MCM-41-type mesoporous silica (Figure 4B).

Small-angle XRD patterns (Figure 4B) demonstrated the obtaining of an ordered hexagonal pore array for all silica-type matrices based on the characteristic Bragg reflections. The introduction of aminopropyl groups influenced the structure of silica, and a shift towards higher *d*-spacing values was noticed in small-angle XRD patterns, especially for SBA-NH_2_ material that had a higher content of organic moieties than MCMB-NH_2_. Though the functionalization conditions of both mesoporous silica samples were identic, a lower content of amino groups was obtained in the case of MCMB-NH_2_ material, probably because the twisted channels of MCM-41B hindered the accessibility of organic moieties to the silica pore walls.

**Figure 5:**
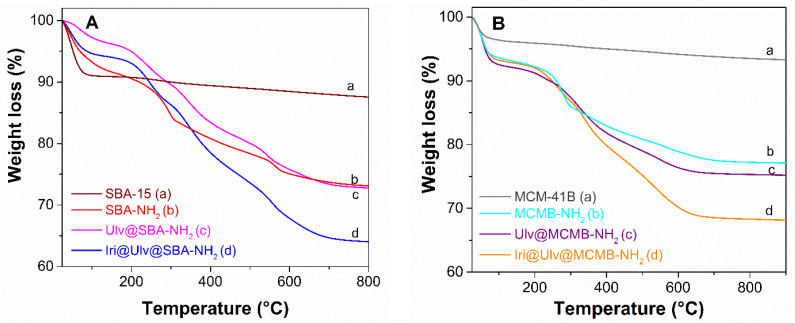
Thermogravimetric analyses for samples based on SBA-15 (silica with large pores) (**A**) and samples containing MCM-41B (silica with small pores) (**B**).

The aminopropyl-modified silica materials presented a decrease in the specific surface area value (984 m^2^/g to 376 m^2^/g in the case of SBA-15-type silica and from 728 m^2^/g to 491 m^2^/g for MCM-41B-type silica), and a significant reduction in pore volume (from 1.31 cm^3^/g to 0.62 cm^3^/g for SBA-15-type materials and from 0.64 cm^3^/g to 0.61 cm^3^/g in the case of MCM-41B-type materials) through functionalization (Supplementary Material, Table S1). A higher content of positively charged amine groups (in the case of SBA-NH_2_) favored the ulvan deposition on the silica surface through electrostatic interactions (Table 2, Figure 1). The natural polymer content in nanoplatforms determined from the thermogravimetric analysis is 6%(w/w) for Ulv@SBA-NH_2_ (Table 2, Figure 5A (d)) and 1.5%(w/w) in the case of Ulv@MCMB-NH_2_ (Table 2, Figure 5B (d)).

The deposition of ulvan on aminopropyl-functionalized silica samples did not change the morphology of silica particles (Supplementary Material, Figure S5).

**Figure 6:**
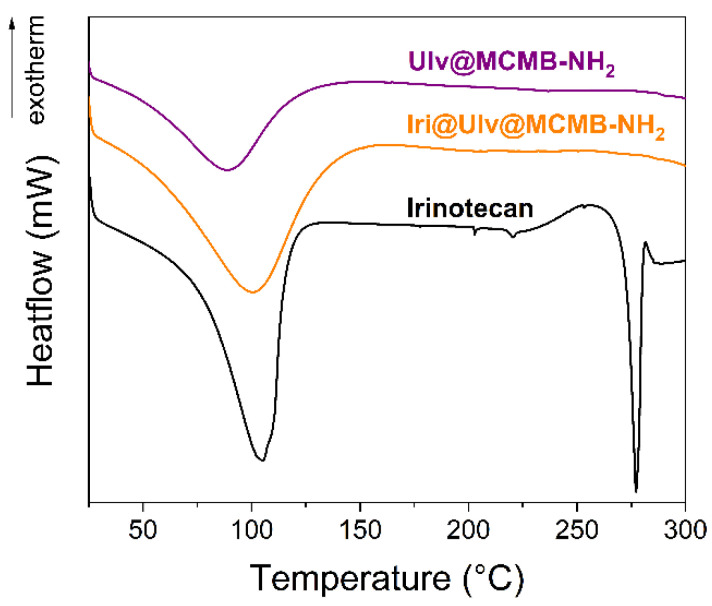
Differential scanning calorimetry analyses of irinotecan, Ulv@MCMB-NH_2_ and Iri@Ulv@MCMB-NH_2_.

Irinotecan was loaded into mesopores of silica in an amorphous state as wide-angle XRD patterns proved (Figure 2b,c). DSC analyses were also performed to assess the physical state of the encapsulated irinotecan (Figure 6). The silica-ulvan nanoplatform, irinotecan-loaded nanoplatform and pure drug exhibit an endothermic effect between 40–140 °C, associated with the evaporation of physiosorbed water. Pure irinotecan melts at 272.5 °C. No endothermic effect associated with the melting of crystalline drug can be noticed for the Iri@Ulv@MCMB−NH_2_, indicating that the anticancer agent is adsorbed only as an amorphous phase.

**Figure 7:**
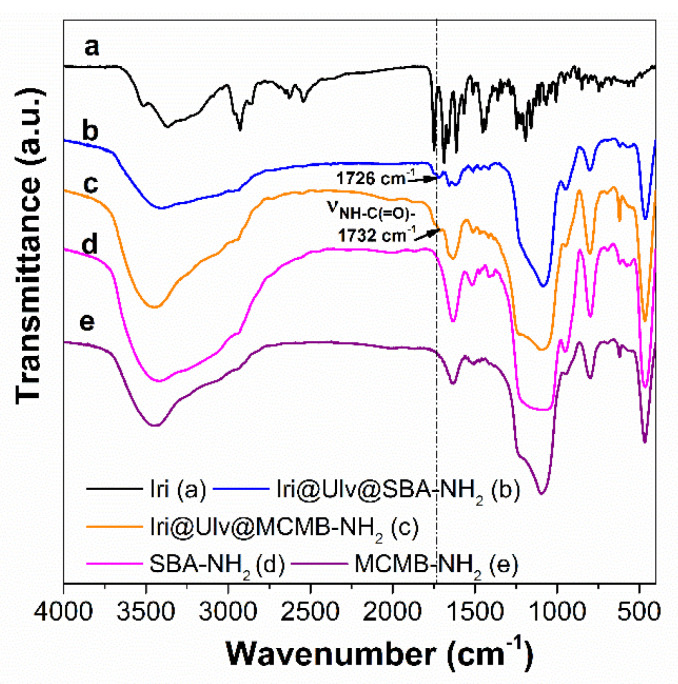
FTIR spectra of irinotecan (**a**); Iri@Ulv@SBA-NH_2_ (**b**); Iri@Ulv@MCMB-NH_2_ (**c**); SBA-NH_2_ (**d**); and MCMB-NH_2_ (**e**).

In the FTIR spectra of irinotecan-loaded silica-ulvan carriers can be seen several bands corresponding to irinotecan and ulvan in the 1400–1200 cm^−1^ domain and 850–600 cm^−1^ region, respectively (Figure 7b,c). The actual amount of irinotecan from ulvan-silica nanoplatforms was determined by thermogravimetric analysis (Figure 5A,B—curves d), which is a suitable technique when the drug loading is performed by the incipient wetness impregnation method [36] or hot-melt loading procedure [31]. This is different than the case of carriers loaded with API by adsorption at equilibrium, when a part of API molecules remains in solution, the drug entrapment efficiency is evaluated by UV-vis spectroscopy [17] or high-performance liquid chromatography [46].

### 3.2. In Vitro Release Experiments of Irinotecan from Silica-Ulvan Nanoplatforms

**Figure 8:**
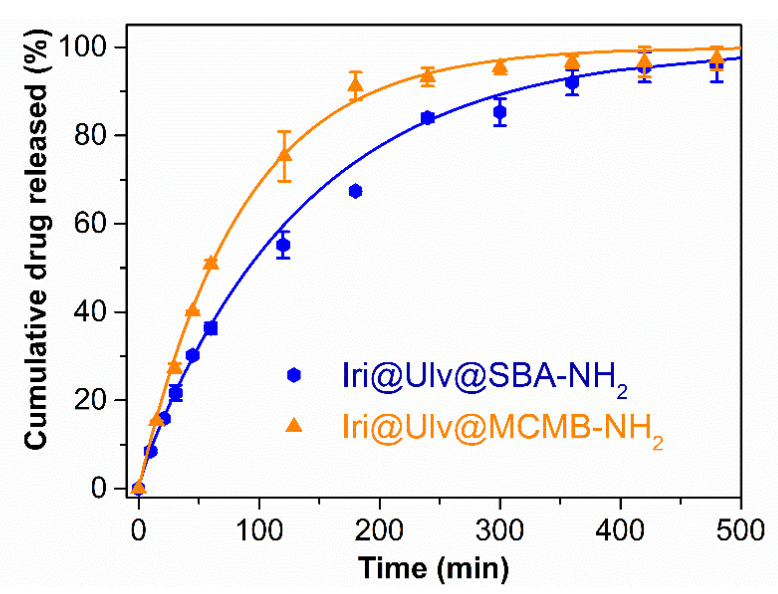
In vitro release profile of irinotecan from silica-ulvan nanoplatforms.

In vitro release experiments of irinotecan from silica-ulvan carriers were performed using an equivalent drug content of 0.1 mg/mL drug in PBS pH 7.6, for 8 h. At set time intervals, aliquots from the release medium were withdrawn and the solution absorbance was read at 255 nm. The experimental data were fitted with the three-parameter model, which considered the adsorption/desorption equilibrium and the diffusion process having first-order kinetics. The free molar energy Gibbs, Δ*G* is proportional to the amount of drug release in the first part of the experiment, while the irinotecan delivery rate is proportional to diffusion constant rate, *k_d_* [47]. The model describes the experimental data (*R*^2^ being in the range of 0.9975–0.9978) for both experiments (Table 3) well. The irinotecan release profile from silica-ulvan carriers are presented in Figure 8.

One can observe that irinotecan is completely released in 8 h from both silica-ulvan nanoplatforms (Figure 8). However, slower release kinetics (ΔG<0) are obtained for the Ulv@SBA-NH_2_ nanoplatform, most likely due to a higher polymer content on the silica surface, which is bound on electrostatic interactions between positively charged amine groups linked to the silica surface and negatively charged sulphonate groups of ulvan. Between irinotecan molecules and amine groups of silica are electrostatic repulsions, which would cause a pronounced burst effect. When silica was coated with polysaccharide, silica amine groups were involved in electrostatic interactions with ulvan sulfonate groups, and hence, the repulsion between drug molecules and silica functional groups was suppressed. This could explain the slower kinetics of irinotecan when Ulv@SBA-15 with a higher amount of polysaccharide was used as the nanoplatform.

The diffusion rate (*k*_d_) is higher for Iri@Ulv@SBA-NH_2_ due to the larger pores of the carrier than in the case of Iri@Ulv@MCMB-NH_2_ (Table 3). Moreover, in the case of the Iri@Ulv@SBA-NH_2_ sample, a higher tendency of drug re-adsorption into the silica pores (*k*_on_ > *k*_off_) was noticed.

### 3.3. Biological Evaluation of Ulvan, Ulvan-Silica Nanoplatforms and Irinotecan-Loaded Samples

#### 3.3.1. Ulvan Biocompatibility Assessment

The ulvan biocompatibility was evaluated based on cellular viability (MTT assay) of normal subcutaneous mouse tissue cells, L929 (ECACC 85011425), after the incubation with several concentrations of polysaccharide aqueous solution.

**Figure 9:**
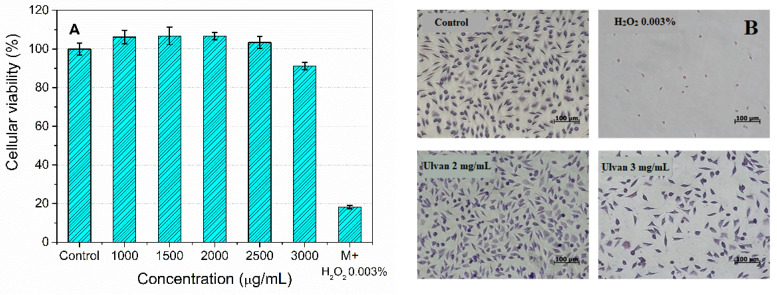
Cell viability of mouse connective tissue fibroblasts (L929 cell line) incubated with ulvan (1000–3000 μg/mL) after 24 h (**A**) and optical micrographs of cells incubated with ulvan after 48 h from the incubation (**B**).

The ulvan extract is biocompatible up to a concentration of 2 mg/mL (Figure 9). A 0.003%(w/w) H_2_O_2_ solution was used as a positive control, which decreased the cellular viability by 20% compared to the control. Cell microscopy images (Figure 9B) of L929 cells stained with Giemsa reagent and treated with polysaccharide extract after 48 h incubation revealed that a 2 mg/mL dose of ulvan did not influence the cell morphology, being similar to control, while a concentration of 3 mg/mL induced a slight cells toxicity (Figure 9A). The ulvan extract exhibited better biocompatibility than that reported by Alves et al., which reported that the sulfonated polysaccharide was not toxic on the same cell line up to a dose of 1 mg/mL [48].

#### 3.3.2. Cytotoxicity Evaluation of Irinotecan-Loaded Nanoplatforms

**Figure 10:**
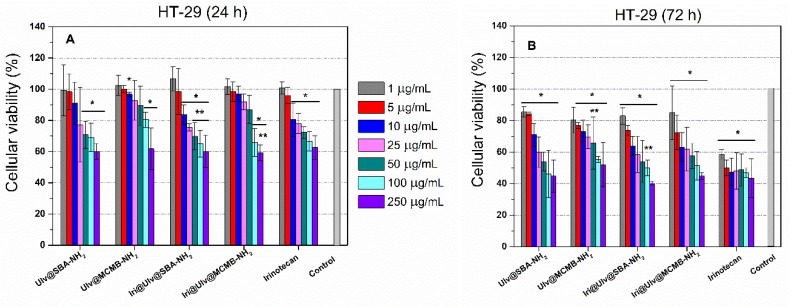
Cytotoxicity evaluation was performed on HT-29 cell line incubated with (1–250 μg/mL) after 24 h (**A**) and 72 h (**B**), respectively (* *p* < 0.05 compared to Control and ** *p* < 0.05 compared to the same concentration of irinotecan—Student *t*-test).

The irinotecan-loaded nanoplatforms were evaluated on a human colorectal adenocarcinoma cell line (HT-29) after 24 h or 72 h of incubation. To compare the results obtained for irinotecan-loaded nanoplatforms with the drug alone, it should be considered that the nanoplatforms contain approximatively 8.5%(w/w) cytostatic agent (Table 2), and hence, the irinotecan concentration tested was at least ten times lower than that of the drug alone. Therefore, the viability of HT-29 cells exposed to 50 µg/mL, 100 µg/mL and 250 µg/mL concentrations of irinotecan-loaded nanoplatforms can be compared with 5 µg/mL, 10 µg/mL and 25 µg/mL of irinotecan. After 24 h, both irinotecan-loaded nanoplatforms exhibited a higher anticancer activity than the drug alone. Though the amount of cytostatic agent in nanoplatforms was at least ten times lower, after 72 h, a similar effect to irinotecan alone was observed. These results could be attributed to a synergistic effect of both silica-ulvan nanoplatforms and irinotecan, as well as to an efficient delivery of cytostatic agents (Figure 8). After 72 h, higher toxicity was observed for all samples, all being significantly different from the control (Figure 10).

**Figure 11:**
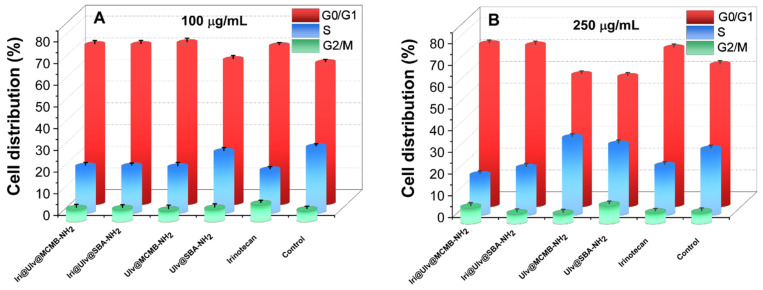
Cell cycle distribution of HT-29 cells after treatment with 100 μg/mL (**A**) and 250 μg/mL (**B**) of samples in the G0/G1, S, and G2/M phases.

The distribution of HT-29 cells over the cell cycle phases was analyzed using flow cytometry, and the results obtained after data acquisition and quantification are shown in Figure 11. The flow cytometry analysis depicted that, after 24 h of exposure to samples at concentrations of 100 and 250 µg/mL, the number of cells in the G0/G1 phase increased, while fewer cells were found in the S phase either for irinotecan or drug-loaded nanoplatforms. One can observe that the cell population in the S phase for cytostatic agent alone and irinotecan-loaded nanoplatforms were reduced in comparison with control. Moreover, the incubation of HT-29 cells in the presence of 250 μg/mL Ulv@SBA-NH_2_ and Ulv@MCMB-NH_2_ resulted in an increase in cell population in the S phase of approximatively 9% and 17%, respectively, over the control, while the G0/G1 phase registered a decrease of only approximatively 7% and 8.5% compared to the control.

## 4. Conclusions

The ulvan extracted from *Ulva lactuca* algae (from Black Sea, Romania), rich in carbohydrates, proved to be biocompatible up to a dose of 2 mg/mL. It was further employed to develop mesoporous silica-ulvan nanoplatforms for irinotecan. To better understand irinotecan release from these nanoplatforms, two types of mesoporous silica were used, one with large pores (SBA-15) and another with smaller pores and ball-like morphology (MCM-41B). A good correlation was achieved between the amount of natural polysaccharide contained on the nanoplatforms and drug release kinetics. A slower delivery of irinotecan was obtained for a higher amount of ulvan in nanoplatforms (the case of Ulv@SBA-NH_2_).

The biological assays evidenced that drug-loaded nanoplatforms exhibited an enhanced anticancer activity on the human colorectal adenocarcinoma cell line (HT-29) than irinotecan alone. The cell cycle analysis proved that when irinotecan was loaded on ulvan-silica nanoplatforms, an increase in the cells number trapped at G0/G1 phase was observed, influencing the development of tumoral cells acting as a cytostatic agent.

## Figures and Tables

**Table 1:** Spectrophotometric assays of methanol and acetone extracts from *Ulva lactuca.*

Solvent	*CHa* (mgCHa/g_extract_)	*CHb* (mgCHb/g_extract_)	*CHT* (mgCHt/g_extract_)	*TPC* (mg GAE/g_extract_)
Methanol	17.1 ± 0.1	25.7 ± 0.2	42.8 ± 0.2	30.4 ± 0.4
Acetone	12.3 ± 0.2	2.9 ± 0.0	15.2 ± 0.2	-

*Cha*—amount of chlorophyll-*a*, *CHb*—amount of chlorophyll-*b*, *CHT*—total amount of chlorophyll, *TPC*—total polyphenolic content, and *GAE*—gallic acid equivalents).

**Table 2:** Silica/organic groups (OG) molar ratio, ulvan content, and irinotecan amount for irinotecan-loaded samples.

Support-Type	Support		Iri@support
	n_SiO2_/n_OG_	Ulv (%w/w)	Irinotecan (%w/w)
Ulv@MCMB-NH_2_	6.25	1.5	8.3
Ulv@SBA-NH_2_	5.06	6.0	8.6

**Table 3:** Kinetic parameters of irinotecan release from ulvan-silica nanoplatforms.

Irinotecan Loaded Ulvan-Silica Nanoplatforms	Three-Parameter Model					Maximum Amount of Drug Released (%)
	Δ*G* (10^21^ J)	*k_d_* (min^−1^)	*k*_off_ (min^−1^)	*k*_on_ (min^−1^)	*R* ^2^	
Iri@Ulv@SBA-NH_2_	−3.36	0.032	0.015	0.034	0.9978	96.1 ± 3.9
Iri@Ulv@MCMB-NH_2_	1.87	0.019	1.501	0.969	0.9976	97.4 ± 2.6

## Data Availability

All the data is available within the manuscript.

## Supplementary Materials

The following supporting information can be downloaded at: https://www.mdpi.com/article/10.3390/ma15197003/s1, Figure S1: DSC analysis of ulvan extract; Figure S2: EDX analysis (A) and SEM image with EDX elemental mapping (B) of polysaccharide extract from *Ulva lactuca*; Figure S3: TEM micrographs of MCM-41B-type silica prepared in the presence of different molar ratio of TEOS/N-BUDEA; Figure S4: N_2_ adsorption-desorption isotherms of MCM-41B-type mesoporous silica prepared in the presence of different molar ratio of TEOS/N-BUDEA (Inset: Pore size distribution curves for MCM-41B type materials); Figure S5. SEM micrographs of Ulv@SBA-NH_2_ and Ulv@MCMB-NH_2_; Table S1: Textural parameters of pristine and functionalized mesoporous silica materials.
