# Supplementary material for: Nanoplatforms for Irinotecan Delivery Based on Mesoporous Silica Modified with a Natural Polysaccharide
Source: Materials (Basel). 2022 Oct 9;15(19):7003. doi: 10.3390/ma15197003 (PMC9571191; doi:10.3390/ma15197003)
Supplement: Supplementary file 1 [file materials-15-07003-s001.zip › materials-1908476-supplementary.pdf]

Nanoplatforms for irinotecan delivery based on mesoporous silica modified with a natural polysaccharide

Ana-Maria Brezoiu[1], Ana-Maria Prelipcean[2], Daniel Lincu[1,3], Mihaela Deaconu[1], Eugeniu Vasile[1], Rodica Tatia[2], Ana-Maria Seciu-Grama[2], Cristian Matei[1], Daniela Berger[1,*]

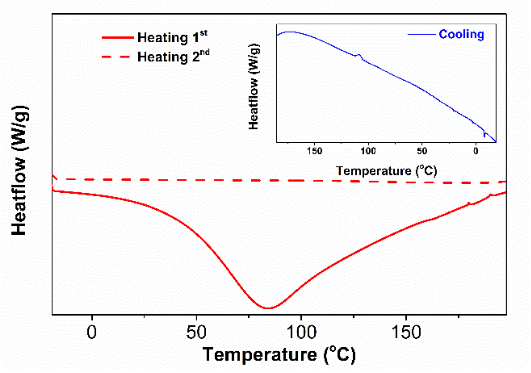

Figure S1. DSC analysis of ulvan extract.

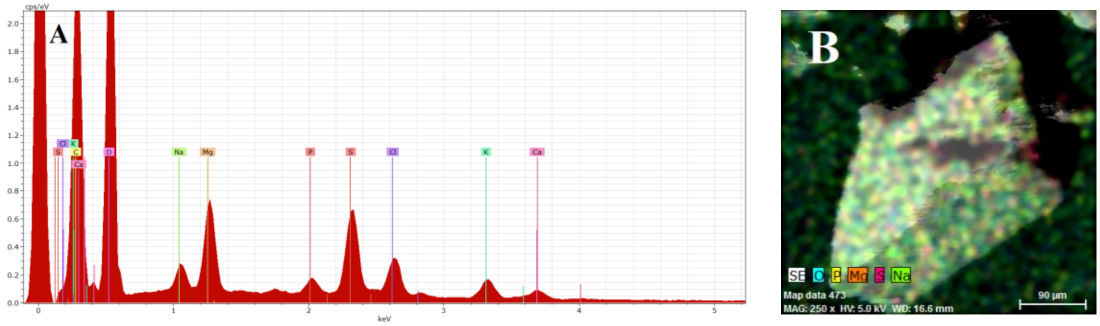

Figure S2. EDX analysis (A) and SEM image with EDX elemental mapping (B) of polysaccharide extract from *Ulva lactuca*.

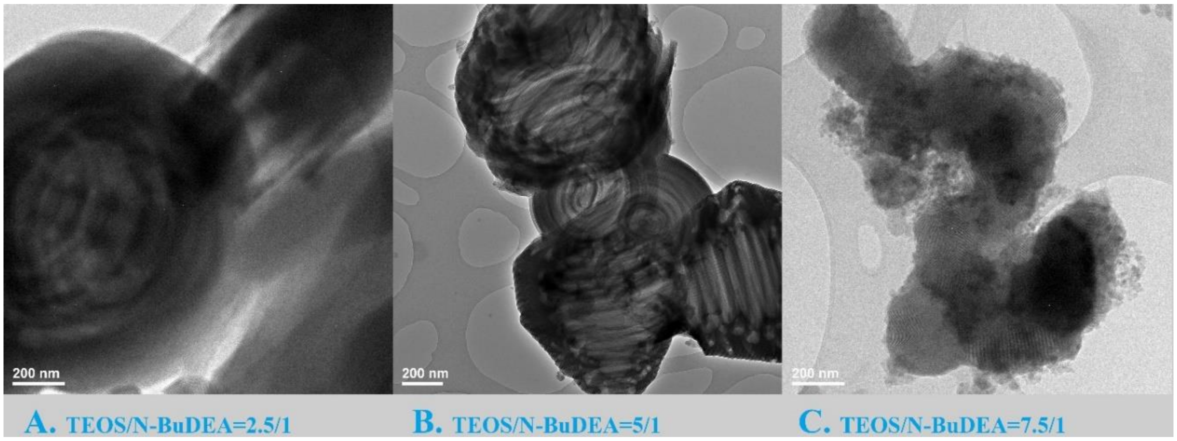

Figure S3. TEM micrographs of MCM-41B-type silica prepared in the presence of different molar ratio of TEOS/N-BUDEA.

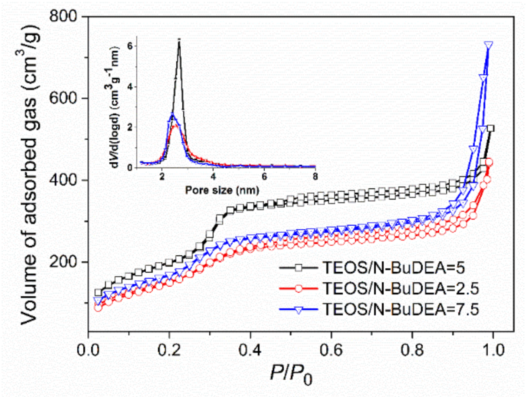

Figure S4. N_2 adsorption-desorption isotherms of MCM-41B-type mesoporous silica prepared in the presence of different molar ratio of TEOS/N-BUDEA (Inset: Pore size distribution curves for MCM-41B-type materials).

Table S1. Textural parameters of pristine and functionalized mesoporous silica materials

Support	TEOS/N-BuDEA	S_{BET} (m²/g)	d_{BJH} (nm)	V_P (cm³/g)
MCM41-B	5.0	728	2.66	0.64
MCM41-B (1)	2.5	595	2.39	0.75
MCM41-B (2)	7.5	613	2.39	0.44
MCMB-NH_2	5.0	491	2.52	0.61
SBA-15	-	984	8.10	1.31
SBA-NH_2	-	376	7.42	0.62

V_P was computed for (d_{pore} < 10 nm), specific surface area, S_{BET}, was determined based on Brunauer-Emmet-Teller method, while the average pore size values were determined by Barrett-Joyner-Halenda model.

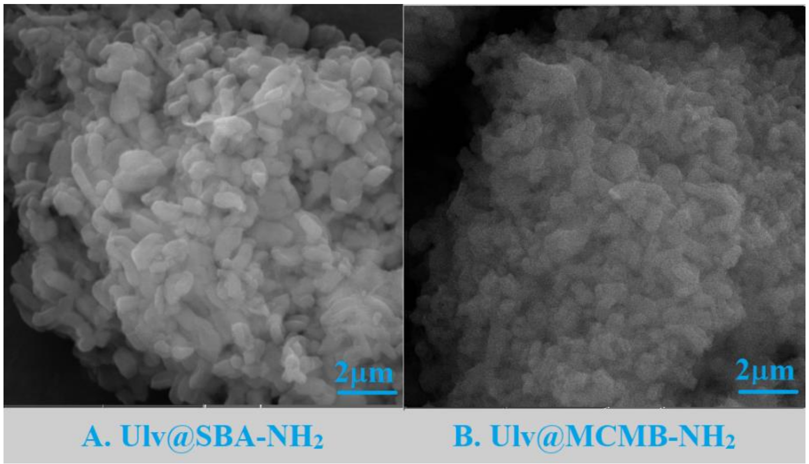

Figure S5. SEM micrographs of Ulv@SBA-NH_2 and Ulv@MCMB-NH_2.
